# Molecular interaction between plants and *Trichoderma* species against soil-borne plant pathogens

**DOI:** 10.3389/fpls.2023.1145715

**Published:** 2023-05-15

**Authors:** Pranab Dutta, Madhusmita Mahanta, Soibam Basanta Singh, Dwipendra Thakuria, Lipa Deb, Arti Kumari, Gunadhya K. Upamanya, Sarodee Boruah, Utpal Dey, A. K. Mishra, Lydia Vanlaltani, Dumpapenchala VijayReddy, Punabati Heisnam, Abhay K. Pandey

**Affiliations:** ^1^ School of Crop Protection, College of Post Graduate Studies in Agricultural Sciences, Central Agricultural University (Imphal), Meghalaya, Imphal, India; ^2^ Director of Research, Central Agricultural University (Imphal), Imphal, India; ^3^ School of Natural Resource Management, College of Post Graduate Studies in Agricultural Sciences, Central Agricultural University (Imphal), Imphal, India; ^4^ Sarat Chandra Singha (SCS) College of Agriculture, Assam Agricultural University (Jorhat), Dhubri, Assam, India; ^5^ Krishi Vigyan Kendra (KVK)-Tinsukia, Assam Agricultural University (Jorhat), Tinsukia, Assam, India; ^6^ Krishi Vigyan Kendra (KVK)-Sepahijala, Central Agricultural University (Imphal), Tripura, Sepahijala, India; ^7^ Department of Plant Pathology, Dr Rajendra Prasad Central Agricultural University, Bihar, Samastipur, India; ^8^ Department of Agronomy, Central Agricultural University (Imphal), Pasighat, India; ^9^ Department of Mycology and Microbiology, Tea Research Association, North Bengal Regional, R & D Center, Jalpaiguri, West Bengal, India

**Keywords:** soil-borne phytopathogen, *Trichoderma*, molecular interaction, disease management, host plant

## Abstract

*Trichoderma* spp. (Hypocreales) are used worldwide as a lucrative biocontrol agent. The interactions of *Trichoderma* spp. with host plants and pathogens at a molecular level are important in understanding the various mechanisms adopted by the fungus to attain a close relationship with their plant host through superior antifungal/antimicrobial activity. When working in synchrony, mycoparasitism, antibiosis, competition, and the induction of a systemic acquired resistance (SAR)-like response are considered key factors in deciding the biocontrol potential of *Trichoderma*. Sucrose-rich root exudates of the host plant attract *Trichoderma*. The soluble secretome of *Trichoderma* plays a significant role in attachment to and penetration and colonization of plant roots, as well as modulating the mycoparasitic and antibiosis activity of *Trichoderma.* This review aims to gather information on how *Trichoderma* interacts with host plants and its role as a biocontrol agent of soil-borne phytopathogens, and to give a comprehensive account of the diverse molecular aspects of this interaction.

## 1 Introduction

Agriculture is an economic activity that deals with the scientific production of crops to address world hunger. Food is a fundamental right of humans; therefore, it is the utmost concern of all countries to increase their agricultural production, as the global population is expected to reach nearly 10 billion by 2050 (Gill and Garg, 2014; Dutta et al., 2022a). However, one of the major challenges encountered by agriculture today is the sustainable production of high-quality food in a sufficient quantity to meet the needs of the producer and consumer. Among the various biotic and abiotic factors contributing to the economic yield loss of crops, destruction due to diseases caused by filamentous fungi is of foremost importance (Singh, 2014). Soil-borne plant pathogens lead to a significant reduction in crop yield by causing diseases such as die-back, wilting, and root rot. They usually target the roots to enter into the plant system and directly influence water and nutrient uptake capacity. Soil-borne diseases therefore have a direct negative impact on plant growth and development (Dignam et al., 2022). The management of soil-borne plant diseases is a cumbersome task. Large amounts of chemical pesticides are administered early in the farming process to counteract these phytopathogens. The use of chemical pesticides, however, has a negative impact on the environment, such as residual toxicity and soil pollution. Therefore, the biological management of plant diseases with different bacterial and fungal biocontrol agents is considered a safer option. *Trichoderma*, a soil-inhabiting ascomycete fungus, is widely used for its versatile plant growth-promoting (PGP) and biocontrol activity. First described by Persoon (1794), the genus *Trichoderma* outraces phytopathogens in the competition for space, nutrients, antibiosis, and mycoparasitism (Mukherjee et al., 2013). Furthermore, *Trichoderma* is also known to colonize plant roots, to enhance plants’ systemic defenses, viz., systemic acquired resistance (SAR) and induced systemic resistance (ISR), and to promote plant growth by modulating the phytohormonal blend. To do so, *Trichoderma* needs to interact and establish a good relationship with the host plant. Proteins or peptides are the communicating molecule in any plant–fungus interaction. Plants and fungi communicate and perceive their surroundings *via* the secretion and perception of different peptides. Understanding the soluble secretome of *Trichoderma* will shed light on the mechanisms of molecular crosstalk between plant roots and *Trichoderma*, and will explain the mechanism behind PGP and biocontrol activities.

## 2 *Trichoderma*: role as a biocontrol agent

**Figure 1:**
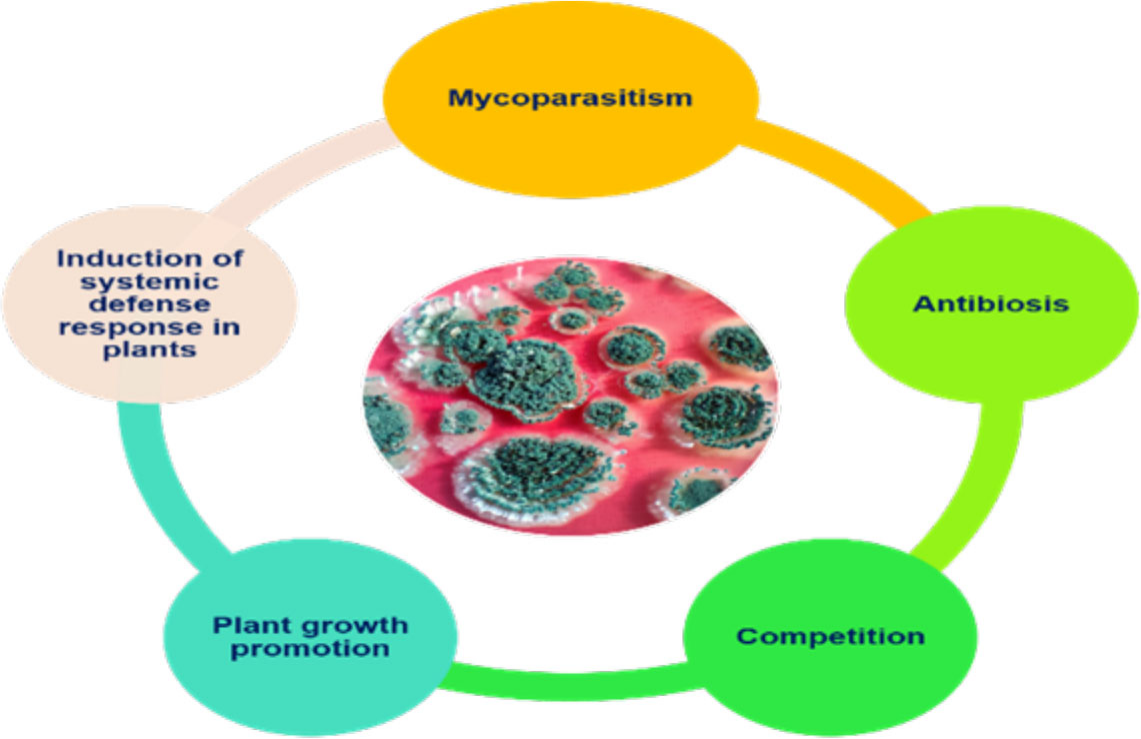
Key components of *Trichoderma*-mediated disease suppression.

In the early 1930s, the biological control potential of *Trichoderma* was realized. Weindling, while working with *T. lignorum* and *Rhizoctonia solani*, observed that the mycelial growth of *R. solani* was inhibited by the profuse mycelial growth of *Trichoderma*. Microscopic observation led to the discovery of a new phenomenon, whereby the hyphae of *T. lignorum* coil around the hyphae of the phytopathogen and penetrate them, which subsequently leads to complete dissolution of the host cytoplasm. The mechanism of parasitization of another fungus by *Trichoderma* was named mycoparasitism by Weindling (1932). The discovery of the mycoparasitic nature of *Trichoderma* led a great volume of work on the subject by many researchers. The studies carried out led to the discovery of different biocontrol mechanisms exhibited by fungi from the genus *Trichoderma*. Genome profiling of three *Trichoderma* species, viz., *T. virens*, *T. atroviride*, and *T. reesei*, by Mukherjee et al. (2012) has opened avenues for understanding the molecular mechanism behind their advantageous biocontrol activities. Mycoparasitism, competition with other soil inhabitants/invaders, and antibiosis are the major modes of action for the biocontrol activity of *Trichoderma* (Zhang et al., 2021). Synchronization between mycoparasitism and antibiosis is necessary for the proper functioning of this biocontrol agent (BCA) (Keswani et al., 2014). Moreover, the host defense activation triggered by *Trichoderma* is a key part of its ability to protect plants against several phytopathogens. Therefore, it can be said that a combination of competitive exclusion, antibiosis, mycoparasitism, and induced systemic resistance (**Figure 1**) is crucial for *Trichoderma*-mediated disease suppression/management (Sharma et al., 2017). *Trichoderma* is a very fast-growing BCA that rapidly colonizes the spermosphere and/or rhizosphere, thereby providing protection to germinating seeds against major soil-borne, seed-borne, and air-borne plant diseases (Mukherjee et al., 2022).

## 3 Major biological control strategies of *Trichoderma*

### 3.1 Mycoparasitism: *Trichoderma*’s deadly weapon for the management of phytopathogens

**Figure 2:**
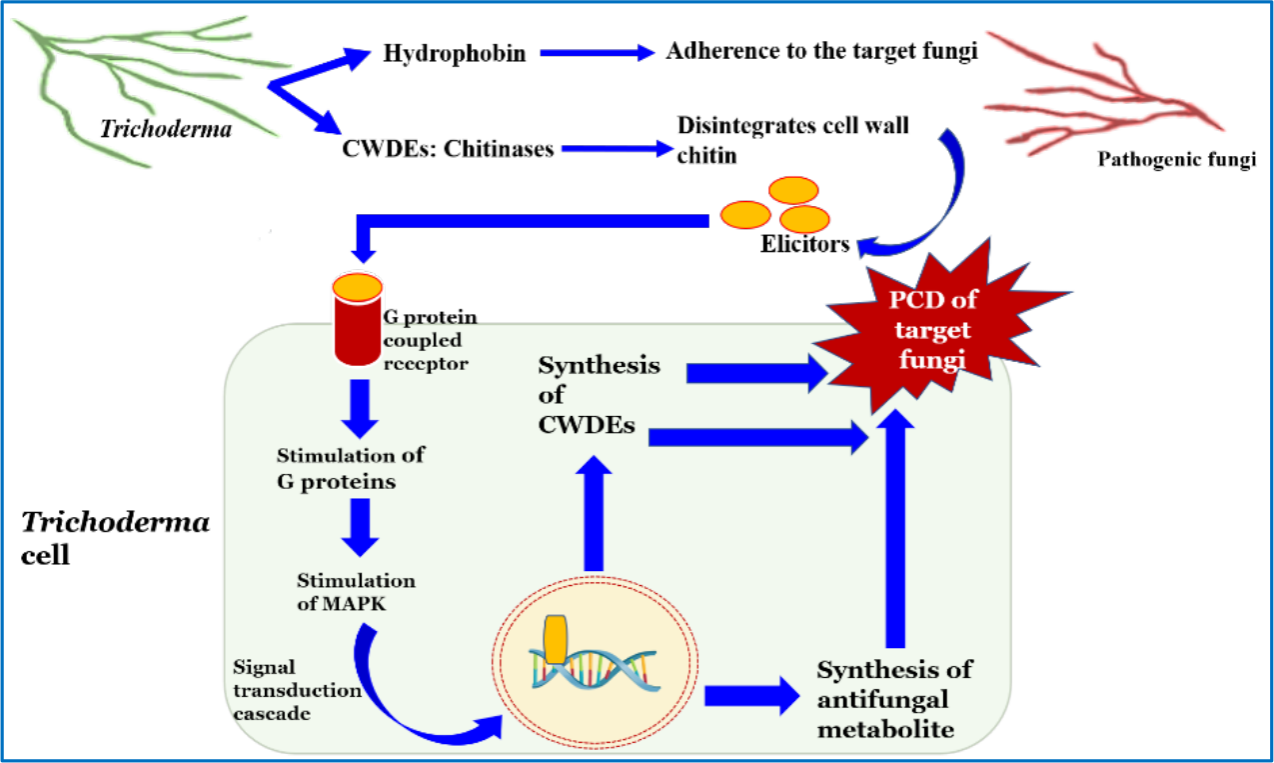
Mode of action of *Trichoderma* against phytopathogens.

The phenomenon of mycoparasitization by *Trichoderma*, as first reported by Weindling (1932), is a complex process involving sequential events. Direct confrontation with the fungal pathogen and the secretion of cell wall-degrading enzymes (CWDEs) is followed by the penetration and subsequent killing of the fungal phytopathogen (Woo and Lorito, 2007). The primary identification and attachment between *Trichoderma* and the prey fungi is mediated by the binding of the cell wall carbohydrates of *Trichoderma* to the lectin of the target fungi, which is followed by hyphal coiling. The adhesion of *Trichoderma* to the mycelium of the host fungi is facilitated by hydrophobins, which is evident from the expression of the *Vel1* gene of *T. virens* encoding hydrophobins (Viterbo and Chet, 2006). Penetration into the target hyphae occurs *via* the development of appressoria containing a high concentration of osmotic solutes such as glycerol, which is necessary for exerting mechanical pressure to invade the hyphal wall. The use of fungitoxic CWDEs, such as chitinases, glucanases, and proteases, by *Trichoderma* combined with the mechanical strength exerted by the appressorium is crucial to the successful penetration of the host hyphae. Following penetration into the lumen of the target hyphae, the cumulative effect of the CWDEs dissolves the host cell wall, which ultimately results in parasitization and facilitates the assimilation of cell wall content, leading to the subsequent killing of the target fungus (Howell, 2003; Harman et al., 2004; Sood et al., 2020). In addition, *Trichoderma* disarms the target fungi by deactivating the enzymes necessary for pathogenic fungi to colonize and penetrate the plant tissue (Harman et al., 2004). There are approximately 75 species of *Trichoderma* reported to exhibit mycoparasitic activity against a wide range of phytopathogens (Verena et al., 2011; Harwoko et al., 2021). Different studies have revealed the significant effect of several strains/species of *Trichoderma* in the management of phytopathogens such as *Fusarium oxysporum*, *F. culmorum*, *Gaeumannomyces graminis* var. *tritici*, *Pythium aphanidermatum*, *R. solani*, and *Sclerotium rolfsii* in both greenhouse and field conditions (Das et al., 2006; Dutta et al., 2008; Gajera et al., 2013). The hyperparasitization of *F. graminearum* by *Trichoderma* takes place by the *Trichoderma* clutching and coiling around the target mycelium, interpenetration, and other mechanisms, resulting in deformed mycelium of *F. graminearum* that eventually disappear (Tian et al., 2018). Chitinase secreted by *T. hamatum* plays an important role in promoting disintegration of fungal cell wall, chitin assimilation, mycelial autolysis, mycoparasitism, and impeding mycelial growth, spore germination, and spore formation (Saravanakumar et al., 2017). Similarly, *T. koningiopsis* exhibits mycoparasitic activity against *S. sclerotiorum* by invading the host hyphae, which it achieves by attaching to and wrapping around the targeted hyphae and then breaking them into small fragments until it completely disintegrates (Shaw et al., 2016). *Trichoderma* perceives the presence of target fungi in its surroundings *via* seven transmembrane G protein-coupled receptors, e.g., Grp1 (Omann et al., 2012). When pathogen ligands bind to the receptor, it causes a downstream signaling cascade by stimulating G proteins and mitogen-activated protein kinases (MAPKs). There are three MAPKs known in different *Trichoderma* species, viz., MAPKKK, MAPKK, and MAPK. Signal transduction *via* these pathways may have an important role in the mycoparasitization and biocontrol of phytopathogens. Furthermore, the synthesis and secretion of pathogenesis-related enzymes, viz., CWDE and fungitoxic secondary metabolites such as peptaibols, is an extremely useful chemical resource used by *Trichoderma* to eradicate pathogens (**Figure 2**) (Omann et al., 2012; Gajera et al., 2013; Dutta et al., 2022b). Although *Trichoderma* spp. are traditionally known as necrotrophic mycoparasites, an extensive scientific study (Mukherjee et al., 2022) has also revealed hemibiotrophic nature. Hemibiotrophic *Trichoderma* causes minor damage to the cell wall of the host fungi and is reported to exist intracellularly for a notable period of time.

#### 3.1.1 Evolution of mycoparasitism from a genomic perspective

The ability of the fungi to grow indefinitely as hyphae with great metabolic diversity and their ability to interact with other living components of the ecosystem account for their evolutionary success (Naranjo-Ortiz and Gabaldon, 2019). Mycoparasitic associations were found in the oldest fungal fossil, aged 410 million years (Hass et al., 1994). Comparison of the genome sequences of *T. atroviride*, *T. reesei*, and *T. virens* revealed information about the common ancestral mycotrophic lifestyle of these species (Kubicek et al., 2011; Schmoll et al., 2016; Karlsson et al., 2017). Their mycotrophic lifestyle further evolved to slowly colonize dead wood, plants, animals, and immunocompromised humans, providing new ecological niches for their growth and development (Druzhinina et al., 2018). It is speculated that the ancestor of *Trichoderma* had limited cellulolytic capacity and fed on either fungi or arthropods, and lateral gene transfer (LGT) is to some extent considered responsible for the formation of this genus. Nearly half the genes for plant CWDEs [belonging to the group called carbohydrate active enzymes (CAZymes)] found in genome profiling of *Trichoderma* were from plant-associated Ascomycota, indicating a competitive advantage for the mycoparasite in colonizing and feeding on these fungi. However, LGT is not reported in *Trichoderma* for the mycoparasitism of unrelated fungi such as basidiomycetes and oomycetes (Druzhinina et al., 2018; Mukherjee et al., 2022). A comparative analysis of the presence in 12 *Trichoderma* spp. of peculiar gene families that are not shared by other fungi might be useful in explaining their ability to deconstruct host/prey cells. An extremely high number of genes gained by *Trichoderma* belong to different domains, viz., heterokaryon incompatibility (HET), ankyrin repeat, and the major facilitator superfamily (MFS) transporter families. In comparison with other fungi, *Trichoderma* genomes have been found to possess a higher number of gene families encoding CAZymes, secondary metabolism-related genes, and transcription factors. Moreover, as well as heterokaryon incompatibility, the HET genes may play a important role in sensing the prey fungus (Kubicek et al., 2019).

The study of the transcriptomics of *Trichoderma*’s interactions with host fungi revealed that the strategies taken up against prey fungi differ among different *Trichoderma* species. Atanasova et al. (2013) conducted a transcriptomic study on the specific interactions of three *Trichoderma* spp., viz., *T. atroviride*, *T. virens*, and *T. reesei*, with *R. solani*, which revealed the different strategies adopted by these BCAs. Th results revealed that *T. atroviride* uses diverse strategies that include the up-regulation of the biosynthesis of secondary metabolites and enzymes such as GH16 β-glucanases, proteases, and small secreted proteins. *T. reesei* was found to increase the expression of cellulases, hemicellulases, and transporter-encoding genes, whereas *T. virens* relied strongly on its toxic secondary metabolite, expressing primarily the genes responsible for gliotoxin biosynthesis. Expression of genes encoding β-1,3- and β-1,6-endoglucanase were observed in the transcriptome of *T. harzianum* during co-culture with host fungi. Similar results were obtained for *T. asperellum* during co-culture with mycophytopathogens (Mukherjee et al., 2022). Further research on transcriptomics revealed the up-regulation of genes encoding proteases in *Trichoderma* transcriptomes, indicating that proteolysis is a prominent process in this BCA’s mycoparasitism (Morán-Diez et al., 2019). Zapparata et al. (2021) studied the interactions of *T. gamsii* with *F. graminearum*, with a special focus on transcriptomic changes in both organisms during the sensing phase. The findings revealed an increase in the expression of genes for ferric reductase in *T. gamsii*, which are essential for iron competition among both fungi; similarly, the expression of defensive genes such as genes encoding killer toxin and transporters was upregulated by *F. graminearum*.

### 3.2 Antibiosis and role of secondary metabolites of *Trichoderma*

Study of the mycoparasitic activity of *T. lignorum* on *R. solani* (Weindling, 1932) *via* coiling of hyphae, coagulation of protoplasts, and loss of vacuolated structures led to the discovery of a lethal principle with the ability to suppress *R. solani* in pot cultures (Weindling and Emerson, 1936; Weindling and Fawcett, 1936). The lethal principle was later identified as gliotoxin—a highly antimicrobial secondary metabolite of *Trichoderma* (Weindling, 1934). *Trichoderma* secretes a wide range of chemically divergent secondary metabolites with broad-spectrum antimicrobial activity in the vicinity of their niche, which in turn inhibit the growth or spore germination of mycopathogens, a process known as antibiosis (Keswani et al., 2014). Owing to their biochemical nature, the secondary metabolites of *Trichoderma* perform antibiosis by acting as the metabolic inhibitor of translational pathways, thereby blocking protein synthesis and promoting mycoparasitism by helping in the penetration of the hyphae of prey fungus, as well as inhibiting cell wall synthesis, growth, reproduction, sporulation, nutrient uptake, and metabolite production by target pathogens. Moreover, the antibiosis mediated by the secondary metabolites of *Trichoderma* is greatly influenced by the species and strain of the fungal agent (Khan et al., 2020). A pot assay targeting *R. solani* and *P. debaryanum* in cucumber and pea revealed that the suppression of targeted pathogens was attributed to the antibiosis mechanism of *T. virens* (Allen and Haenseler, 1935). Diverse studies conducted since then have revealed considerable antifungal/antimicrobial activities exhibited by several secondary metabolites of *Trichoderma* against a wide range of phytopathogens (**Table 1**). Viridiol is another antifungal compound released by *Trichoderma* spp. that also inhibits the enzyme activity of 5′-hydroxyaverantin dehydrogenase, which is necessary for aflatoxin biosynthesis in *Aspergillus flavus* and *A. parasiticus*, and, therefore, affects aflatoxin biosynthesis (Sakuno et al., 2000; Wipf and Kerekes, 2003). In addition to antibiosis, these metabolites could also play a significant role in competition, mycoparasitism, and stimulating the plant’s immune system (Zeilinger et al., 2016). It is, therefore, very difficult to study mycoparasitism in isolation. A comparative assessment of sclerotial parasitism, hyphal parasitism, and antibiosis exhibited by *T. virens* (P strain), led Mukherjee et al. (1995), concluded that sclerotial parasitism is the major mechanism used in controlling *S. rolfsii* and *R. solani* in soil (Dutta et al., 2013; Dutta, 2018). In a mutant of *T. virens* (developed using gamma ray-induced mutagenesis) with upregulated genes for plant interaction, the production of secondary metabolites was found to provide excellent protection against collar rot in lentil and chickpea in both greenhouse and on-farm trials (Mukherjee et al., 2019). A UV-induced mutant of *T. virens* deficient in genes for mycoparasitism was observed to be an equally efficient biocontrol agent against *R. solani* as its parental type, and gliotoxin-deficient mutants exhibited a similar result (Howell, 1987; Howell, 2003); however, sclerotial parasitism remained unexamined by these experiments. Therefore, this case raised the question of the mechanism used by *Trichoderma* for efficient control of phytopathogens, and the probable role of induced systemic resistance in host plant was emphasized. Tu (1980) observed that the sclerotia of *Sclerotinia sclerotiorum* were readily parasitized by *T. virens*, with extensive hyphal growth of the mycoparasite inside the colonized sclerotia; however, they could not find any conidia inside. A study conducted by Liu et al. (2009) revealed that, during symbiotic colonization of plant roots by *Trichoderma*, *Trichoderma* secretes a higher number of secondary metabolites, such as pachybasin and chrysophanol, with a lower degree of oxidation and less antimicrobial activity. Interestingly, when the host plant is encountered by any phytopathogens, the reactive oxygen species (ROS) released by the plant convert these weakly antimicrobial metabolites to highly antimicrobial oxidized secondary metabolites, viz., 1,5-dihydroxy-3-hydroxymethyl-9,10-anthraquinone, 1,7-dihydroxy-3-hydroxymethyl-9,10-anthraquinone, and emodine. They perform dual beneficial roles by both acting as a powerful antifungal agent that helps in promoting the competitive efficiency of *Trichoderma* and escalating the immune response of the host plant to other phytopathogens. This finding indicates that, in a tripartite interaction of plant–*Trichoderma*–pathogen, the plant has been previously well equipped with a reservoir of anthraquinone secondary metabolites of *Trichoderma* that engage in antagonistic activity only when the plant encounters a pathogenic invasion. A study of the genomics of *T. virens* revealed that *NRPS Tex2* (non-ribosomal peptide synthetase-encoding gene Tex2) is responsible for the assemblage of 11- and 14-module peptaibols (Mukherjee et al., 2011), which elicit strong antimicrobial effects. Trichokonin VI, a peptaibol isolated from *T. pseudokoningii*, is reported to cause programmed cell death in *F. oxysporum via* formation of voltage-gated channels in the pathogen membrane. Similarly, *SMF2*-derived trichokonin VI in *T. pseudokoningii* was reported to exhibit antimicrobial activity against wide range of fungal phytopathogens by stimulating wide-ranging apoptotic programmed cell death (PCD) (Tijerino et al., 2011; Shi et al., 2012; Sood et al., 2020). Gliotoxin and gliovirin are the polyketides synthesized by the P and Q group strains of *Trichoderma* and have a significant role in managing deadly soil-borne phytopathogens. Interestingly, the *T. virens* P group strain is highly antagonistic to *P. ultimum*, but not to *R. solani*. Similarly, the Q group strain adversely affects *R. solani* (Howell et al., 2000). Further research into the genomic perspective of the secondary metabolism of *Trichoderma* revealed that the *T. virens* gene *veA* ortholog *vel1* encoded the VELVET protein, which is responsible for regulation of both the biosynthesis and biocontrol activity of gliotoxin, and also adjusts the expression of other genes involved in the secondary metabolism (Mukherjee et al., 2012).

**Table 1:** Diverse secondary metabolites secreted by *Trichoderma* spp. and their functions in plant disease management.

Class of secondary metabolite	Name of the compound	Activity performed	Reference
i) Against bacterial phytopathogens			
Peptaibols	Trichokonin VI, VII, and AVIII	Highly effective against the Gram-positive bacterial phytopathogen *Clavibacter michiganensis* subsp. *michiganensis*, causing bacterial wilt and canker in tomato, potato, and maize	Song et al. (2006)
Pyridone	Trichodin A	Antibiotic activity against Gram-positive bacteria	Wu et al. (2014)
Viridiofungin	Viridiofungin A	Effective against *Erwinia amylovora* and *C. michiganensis*	El-Hasan et al. (2009)
–	Secondary metabolites obtained from *T. pseudoharzianum* (T113) and *T. viridae*	Effective against bacterial phytopathogens, viz., *Ralstonia solanacearum* and *Xanthomonas campestris*	Khan et al. (2020)
ii) Against fungal phytopathogens			
Pyrones	6-Pentyl-2H-pyran-2-one	Antifungal activity against *Rhizoctonia solani* and *Fusarium oxysporum* Effective in reducing *Botrytis* fruit rot of kiwi fruits	Scarselletti and Faull (1994); Poole et al. (1998)
	Viridepyronone	Exhibits 90% antagonistic activity against *Sclerotium rolfsii* at MIC 196 mg/ml	Hill et al. (1995); Evidente et al. (2003); Kishimoto et al. (2005)
	Massoilactone and δ-decanolactone	Effective against *Phytophthora* and *Botrytis*	Hill et al. (1995)
Koninginins	Koninginins A, B, D, E, and G	Antifungal activity against *Gaeumannomyces graminis* var. *tritici*	Almassi et al. (1991); Ghisalberti and Rowland (1993)
	Koninginins A, B, and D	Broad antifungal activity against several fungal phytopathogens, viz., *F. oxysporum*, *Bipolaris sorokiniana*, *Phytophthora cinnamomi*, and *Pythium middletonii*	Dunlop et al. (1989); Chen et al. (2015)
Steroids	Stigmasterol	Antifungal activity against *R. solani*, *S. rolfsii*, *Macrophomina phaseolina*, and *F. oxysporum*	Ahluwalia et al. (2014); Ahluwalia et al. (2015)
	Ergosterol, 3,5,9-trihydroxyergosta-7,22-dien-6-one	Effective against *Pyricularia oryzae*, *Aspergillus niger*, and *Alternaria alternata* at MIC 32 µg/mL	Xuan et al. (2014)
Furanosteroids	Viridin	Broad spectrum antifungal activity against *A. niger*, *Botrytis allii*, *Colletotrichum lini*, *F. caeruleum*, *Stachybotrys atra* and *P. expansum*	Brian and McGowan (1945)
Pyridone	Harzianopyridone	Antagonists to *Botrytis cinerea*, *G. graminis* var. *tritici*, *R. solani*, *Phytophthora* spp., *Leptosphaeria maculans*, *S. rolfsii* and *F. oxysporum*	Dickinson et al. (1989); Vinale et al. (2006); Vinale et al. (2009); Ahluwalia et al. (2015)
	Harzianic acid	Highly antifungal activity against soil-borne plant pathogens such as *Pythium irregulare*, *Sclerotinia sclerotiorum*, and *R. solani*	Vinale et al. (2009)
Epipolythiodioxopiperazines	Gliotoxin	Inhibitory against *Rhizoctonia bataticola*, *M. phaseolina*, *Pythium debaryanum*, *Pythium aphanidermatum*, *S. rolfsii* and *R. solani*,	Jones and Pettit (1987); Singh et al. (2005)
	Gliovirin	Antagonistic activity against *Pythium ultimum* and *R. solani*	Howell and Stipanovic (1983); Nakano et al. (1990)
Peptaibols	Trichokonins VI, VII, and VIII	Highly antagonistic to soil-borne phytopathogens such as *R. solani*, *F. oxysporum*, *Verticillium dahliae*, and *B. cinerea*, and *Phytophthora parasitica*	Song et al. (2006); Shi et al. (2012); Zhao et al. (2018)
	Trichokonin	Induces ROS production, synthesis, and accumulation of phenolic compounds at the application site, and activation of multiple defense signaling pathways in plants	Luo et al. (2010)
	Trichorzianines A1 and B1	Exhibit antifungal activity by acting as an inhibitor of spore germination and hyphal elongation of phytopathogenic fungi	Goulard et al. (1995); Lee et al. (1999)
	A-aminoisobutyric acid and isovaline	Highly effective against oomycetes fungi, act as an inhibitor of β-glucan synthase	Dutta et al., (2022b)
	Trichostromaticins A–E	Antagonistic activity against *Moniliophthora perniciosa*, a causal agent of witches’ broom disease in cocoa	Degenkolb et al. (2008)
	Trichogin GA IV and its derivatives	Preventive efficacy against *B. cinerea* infection in tomato plants	Baccelli et al. (2022)
Butenolides	Harzianolide	Antagonistic to pathogens that cause take-all in wheat, viz., *G. graminis* var. *tritici*, *P. ultimum*, *R. solani*, and *B. cinerea*	Almassi et al. (1991); Vinale et al. (2006); Vinale et al. (2009)
	T39butenolide	Highly antagonistic to *G. graminis* var. *tritici*, inhibitory to *P. ultimum*, *R. solani*, and *B. cinerea*	Almassi et al. (1991); Vinale et al. (2006); Vinale et al. (2009)
	5-Hydroxyvertinolide	Antifungal activity against *Mycena citricolor*, the causal organism of American leaf spot disease in coffee	Andrade et al. (1992)
Azaphilones	T22azaphilone, harziphilone, fleephilone,	Antagonistic activity against *P. ultimum*, *G. graminis* var. *tritici*, *R. solani*, *B. cinerea*, *P. cinnamomi*, and *L. maculans.* Provides self-protection to ROS liberated during mycoparasitic interaction with *F. oxysporum* f. sp. *Cubanse* race 4	Vinale et al. (2006); Vinale et al. (2008); Vinale et al. (2009); Pang et al. (2020)
Koninginins	Koninginins A-E	Inhibitory to *G. graminis* var. *tritici;* antifungal activity against *Alternaria panax*, *B. sorokiniana*, *F. oxysporum*, *F. solani*, *P. cinnamomi*, and *P. middletonii*	Almassi et al. (1991); Dunlop et al. (1989); Ghisalberti and Rowland (1993); Chen et al. (2015)
Anthraquinones	1,8-Dihydroxy-3-methylanthraquinone, 1-hydroxy-3-methylanthraquinone	Exhibit antifungal activity against *G. graminis* var. *tritici* and *P. ultimum*	Vinale et al. (2006)
	Chrysophanol, pachybasin	Reduced antimicrobial activity; released in symbiotic interaction with plant roots	Liu et al. (2009)
	1,5-Dihydroxy-3-hydroxymethyl-9,10-anthraquinone; emodin; 1,7dihydroxy-3-hydroxymethyl-9,10-anthraquinone	Powerful antimicrobial agent, effective against *R. solani* and *B. cinerea* Escalates host plant’s defense response against phytopathogen	Liu et al. (2009)
Lactone	Cremenolide	Antagonistic activity against *R. solani*, *B. cinerea*, *and F. oxysporum;* exhibit PGP activity	Vinale et al. (2016)
	Aspinolide C	Exhibit antibiotic activity against *B. cinerea* and *Fusarium sporotrichioides;* activates host plant defense against phytopathogenic fungal invasion	Malmierca et al. (2015)
	Cerinolactone	Strong antifungal activity against *Rosellinia necatrix—*the causal agent of white root rot in apple, pear, apricot, strawberry, etc.	Vinale et al. (2012); Arjona-Girona et al. (2014)
	Nafuredin C, nafuredin A	Exhibit moderate antifungal activity	Zhao et al. (2020)
Trichothecenes	Trichodermin	Broad antifungal activity against several phytopathogenic fungi, such as *B. cinerea*, *Colletotrichum lindemuthianum*, *Colletotrichum gloeosporioides*, *Cochliobolus miyabeanus*, *F. oxysporum*, *R. solani*, and *Thanatephorus cucumeris*	Shi et al. (2009); Sha et al. (2013); Shentu et al. (2014)
	Trichodermarins G–N, trichodermol, trichodermin, trichoderminol, trichodermarins A and B, and 2,4,12-trihydroxyapotrichothecene	Exhibit antifungal and antimicrobial activity; highly effective against *B. cinerea*, *C. miyabeanus*, *F. oxysporum* f. sp. *cucumerium*, *F. oxysporum* f. sp. *niveum*, and *Phomopsis asparagi*	Shi et al. (2020)
	Trichobreols A–E	Exhibit broad antifungal activity	Yamazaki et al. (Yamazaki et al., 2020a and Yamazaki et al., 2020b)
	Trichothecinol A, 8-deoxy-trichothecin, trichothecinol B, and trichodermene A	Antagonistic activity against a broad range of soil-borne phytopathogens	Du et al. (2020)
Isocyanide	Dermadin	Antibiotic activity against *Phytophthora* spp.	Dutta et al., (2022b)
Polyketides	Trichoharzianol	Antifungal activity against *Colletotrichum gloeosporioides*	Jeerapong et al. (2015)
Peptide	Alamethicin	Activation of plant defense, viz., ISR and SAR in lima bean	Engelberth et al. (2001)

MIC, minimum inhibitory concentration; PGP, plant-growth promoting.

### 3.3 Competitive exclusion of phytopathogens by *Trichoderma*

*Trichoderma* is known as an aggressive colonizer of plant roots that competes for space, nutrients, water, or oxygen by mobilizing immobile soil nutrients, thereby eliminating other micro-organisms that inhabit their niche (Elad et al., 2000; Dutta et al., 2022b due to the diversified composition of root exudates secreted by plants. Competition among micro-organisms is a strategy to utilize the nutrient hotspots present in the rhizosphere by eliminating other competitors (Guzmán-Guzmán et al., 2023). Therefore, to be an effective colonizer of plant roots, those organisms must have metabolic versatility and the competitive capacity to occupy the nutrient hotspots. In this regard, *Trichoderma* can be considered as an aggressive competitor because it has the capacity to secrete a plethora of chemically diverse secondary metabolites that have an antagonistic effect on other micro-organisms (i.e., competitive capacities) and it also exhibits rapid growth and colonization strategies (indicating metabolic versatility) that enable it to occupy space in rhizosphere, enhance plant growth, and restrict further growth of potentially pathogenic micro-organisms (Saravanakumar et al., 2017). The presence of ATP-binding cassettes transporters (ABC transporters) in *Trichoderma* ensures enhanced competitive ability by conferring resistance to toxic metabolites secreted by other micro-organisms (Harman et al., 2004). Moreover, *Trichoderma* is compatible with sublethal doses of chemical fertilizers such as urea and muriate of potash, and many chemical pesticides such as thiamethoxam, methomyl, imidacloprid, and methyl bromide, which is attributed to the presence of ABC transporters in *Trichoderma* (Chet et al., 1997; Gajera et al., 2013; Dutta et al., 2017). *Trichoderma* releases certain iron chelators, i.e., siderophores, which become bound to iron present in soil. Iron is a key micronutrient for the viability of fungi, and therefore the release of iron-chelating siderophores by *Trichoderma* is detrimental to the growth of other fungi. This is one of the main reasons for the biocontrol potential of *Trichoderma* against soil-borne phytopathogens such as *Pythium*, *Fusarium*, and *Botrytis*, which is inversely proportional to the concentration of nutrients in soil (Tjamos et al., 1992). The discovery of Gtt1 (high-affinity glucose transporter) in *T. harzianum* CECT 2413 raised questions about the probable role of glucose transporters during competition by *Trichoderma*. Delgado-Jarana et al. (2003) observed that *Gtt* gene expression is upregulated when *T. harzianum* CECT 2413 is subjected to growth in nutrient-deficient media. Moreover, a mutant of *Trichoderma* with an additional copy of the glucose transporter gene performed strongly, with a two- to threefold increase in glucose uptake. Glucose metabolism is essential in the assimilation of enzymes and permeases, as well as proteins involved in membrane and cell wall modifications (Delgado-Jarana et al., 2003).

### 3.4 Impact of *Trichoderma* colonization on plant defense and growth promotion

The sessile lifestyle of plants depends on their ability to adapt to the challenges presented by the outside environment in terms of pathogen attack, nutrient starvation, and exposure to toxins and contaminants which are detrimental to its growth. Owing to the abiotic and biotic stresses faced by plants, growth–defense trade-offs take place, which prioritize the acquisition and use of resources (Hacquard et al., 2016). The production of ROS under such conditions determines important developmental processes and cross-kingdom relationships (Segal and Wilson, 2018).

**Figure 3:**
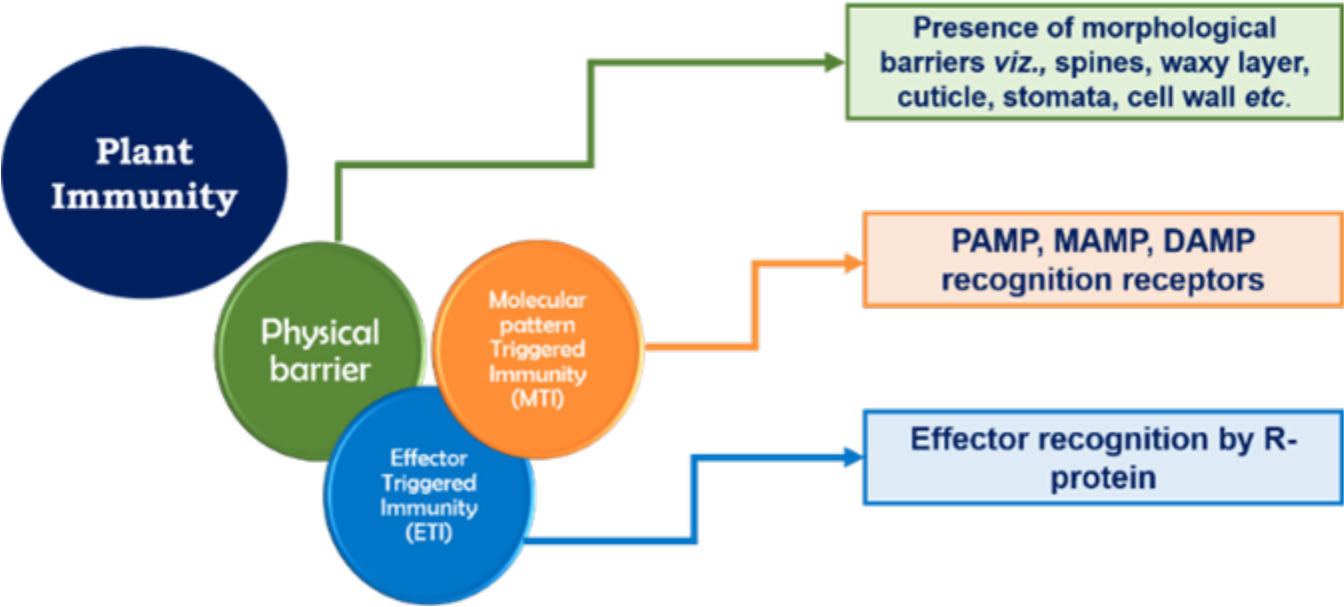
Layers of plant immunity.

Plant immunity consists of a robust three-layered protection (**Figure 3**). The first layer of defense safeguards them from foreign invasion and takes the form of physical barriers such as wax, a cuticle layer, stomata, and the cell wall (Lawry, 2016). The second and third layers are based on molecular pattern recognition. All micro-organisms, irrespective of whether they are pathogenic or beneficial, possess unique molecular patterns known as microbe-associated molecular patterns (MAMPs). The unique molecular patterns present in pathogenic microbes are known as pathogen-associated molecular patterns (PAMPs). The second layer of plant immunity consists of different enzymes and pattern recognition receptors (PRRs) that recognize the MAMPs/PAMPs, which leads to the activation of active immune responses. Another possible element of this layer of plant immunity is damage-associated molecular pattern (DAMP) recognition. DAMP recognition receptors help the plant in recognizing any damage caused to the plant system due to invasion by a micro-organism. Therefore, the innate or basal plant immunity comprises three components, viz., MAMP, PAMP, and DAMP recognition receptors, and together this layer of plant immunity is known as molecular pattern-triggered plant immunity (MTI) (Jones and Dangl, 2006; Benedetti et al., 2015). The third layer is based on effector recognition and is known as effector-triggered immunity (ETI). Effectors (previously known as avirulence or Avr proteins) are the molecules released by pathogens/micro-organisms to escape the MTI of plants. Effectors released by micro-organisms help them to counter MAMP-triggered immunity by, for example, scavenging MAMPs, degrading proteases released by plant, and/or deregulating the primary and secondary signaling pathways of plant host. Containing a nucleotide-binding site and leucine-rich repeats (NBS-LRR), the resistance protein (R protein) present in the plant responds to the effectors and triggers a systemic resistance response (i.e., SAR) due to the accumulation of salicylic acid. However, the effector-triggered interaction is always under a tremendous selection pressure that would enable the pathogen/micro-organism to overcome plant immunity and the plant host to retain its immunity (Lawry, 2016). These two layers of plant immunity (viz., MTI and ETI) greatly influence the plant’s response to invading microbes and trigger strong systemic resistance reactions in the plant. Therefore, in order to successfully enter the plant roots and colonize them, *Trichoderma* need to breach these layers of plant immunity by establishing molecular dialogs with the host plant.

**Figure 4:**
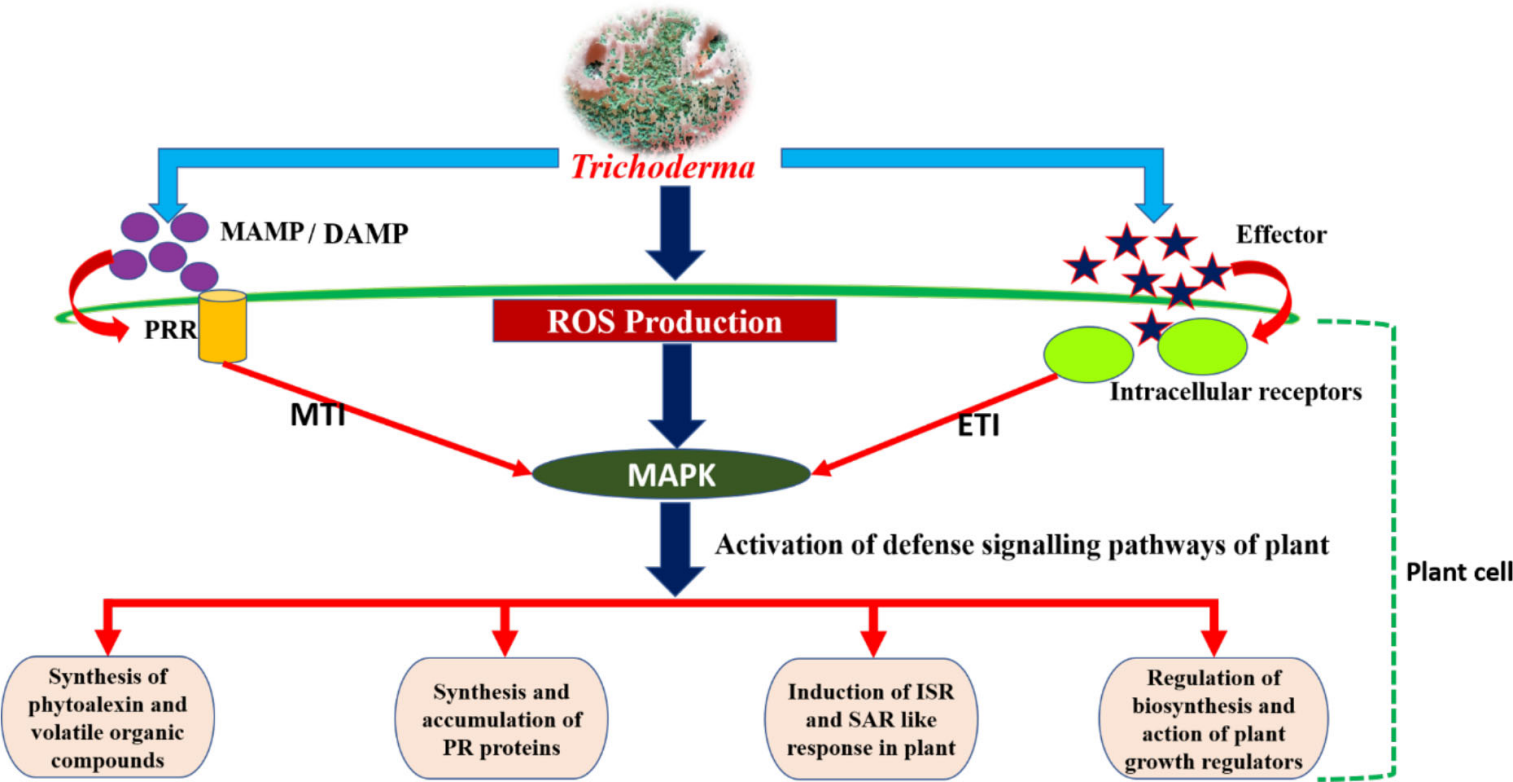
Pictorial representation of plant–*Trichoderma* interaction.

The soil-inhabiting fungi *Trichoderma* are mainly found to be root colonizers. They can establish themselves in the plant as an endophyte after a process of molecular crosstalk that brings a plethora of positive changes for the host. The sucrose-rich plant root exudates act as an attractant for *Trichoderma*, causing root colonization by *Trichoderma*, which activates the plant defense responses and enhances leaf photosynthesis (Vargas et al., 2009). The colonization of plant roots involves *Trichoderma*’s ability to recognize and adhere to roots, penetrate them, and withstand the toxic metabolites produced by the plant in response to invasion. Activation of plant defense takes place through MTI and ETI, which leads to the production of ROS such as H_2_O_2_, ${\text{O}}_{2}^{-}$ , and hydroxyl radical. The ROS further act as a signaling molecule in signal transduction *via* mitogen-activated protein kinase (MAPK), thereby stimulating different pathways of plant defense, such as activation of the phenylalanine ammonia lyase (PAL) enzyme, which is essential in phytoalexin production and synthesis and the accumulation of pathogenesis-related (PR) proteins, and activates the host’s defense responses (Mendoza-Mendoza et al., 2017; **Figure 4**). NADPH oxidase (Nox) is the key enzyme that regulates the production of ROS. Studies have revealed that Nox proteins, particularly NoxR and Nox1, greatly influence the molecular dialog between plant roots and *Trichoderma* during their interaction (Villalobos-Escobedo et al., 2020). The defense responses exhibited by plants to any microbial invasion are energy consuming, and are expressed at the cost of the plants’ own growth and development. Therefore, *Trichoderma* elicits plant growth and development alongside the induction of a strong immune response in plants (Hermosa et al., 2013). In this context, the Nox protein plays a significant role. In a study conducted with a *Trichoderma atroviride* mutant expressing the NoxR protein, co-culture with *Arabidopsis* produced a decrease in feeder root proliferation and phytostimulation when compared with the wild-type strain. However, this also caused an exacerbated response of jasmonic acid-mediated systemic resistance response in the plant when compared with treatment with wild-type *T. atroviride* (Villalobos-Escobedo et al., 2020). Reduction in lateral growth and development in plants when cultured with a *Trichoderma* NoxR mutant may be due to the overactivation of jasmonic acid-mediated responses, leading to a shortage of carbon/other energy resources in the plant that are required for the development of lateral root primordia (Guo et al., 2018).

Invasion and colonization by *Trichoderma* lead to the synthesis and accumulation of different phytohormones, viz., salicylic acid (SA), jasmonic acid (JA), and ethylene (ET). Due to their ability to modulate plant immune responses, these phytohormones are known as the central players of plant defense. The timing, composition, and quantity of the phytohormonal blend produced by plants in response to microbial invasion is greatly influenced by the strain, time, and/or inoculum concentration of the microbe (Pieterse et al., 2009). A study conducted with cucumber and *Trichoderma* in a hydroponic system by Segarra et al. (2007) revealed that, 4 h post inoculation of cucumber roots with *Trichoderma*, the plants exhibited a SAR-like response *via* up-regulated activity of peroxidase and SA. Furthermore, application of a higher inoculum density of *Trichoderma* induced a systemic increase in SA and JA levels within the plant system. This may be due to the expression of ETI causing oxidative bursts in the plant cells, leading to a hypersensitive response in the plant system that ultimately results in the activation of SAR *via* an SA-mediated pathway. These defense responses of plants due to the activation of SA-dependent pathways can be overcome by *Trichoderma* by increasing the JA/ET and auxin responses in plant, which can act as antagonists to SA (Hermosa et al., 2012). When *Trichoderma* was inoculated into the roots of the model plant *Arabidopsis* by Morán-Diez et al. (2012), a decrease in plant defense mediated by SA and JA was observed after 24 h; however, over a longer period, the level of plant defense increased both locally and systematically. They suggested that the lower level of plant defense during the initial 24 h of *Trichoderma* inoculation could be because the plant did not consider the *Trichoderma* as hostile at that point in time. Over time, *Trichoderma* colonizes the epidermal and cortical cells of roots, which plants perceive as a threat, and subsequently the plants try to limit *Trichoderma* from entering into their vascular system by activating plant defenses both locally and systematically *via* upregulation of genes mediating ISR and SAR. Therefore, it can be said that the SA-mediated response of plants is essential in limiting the root colonization by *Trichoderma* to the first two layers of root cortical cells, preventing further invasion into the vascular system (Segarra et al., 2007). Moreover, the ability of *Trichoderma* strains to withstand the highly oxidizing, toxic environment created within the plant system is also a determinant of the degree to which they are effective in colonizing the plant roots (Chen et al., 2011).

**Figure 5:**
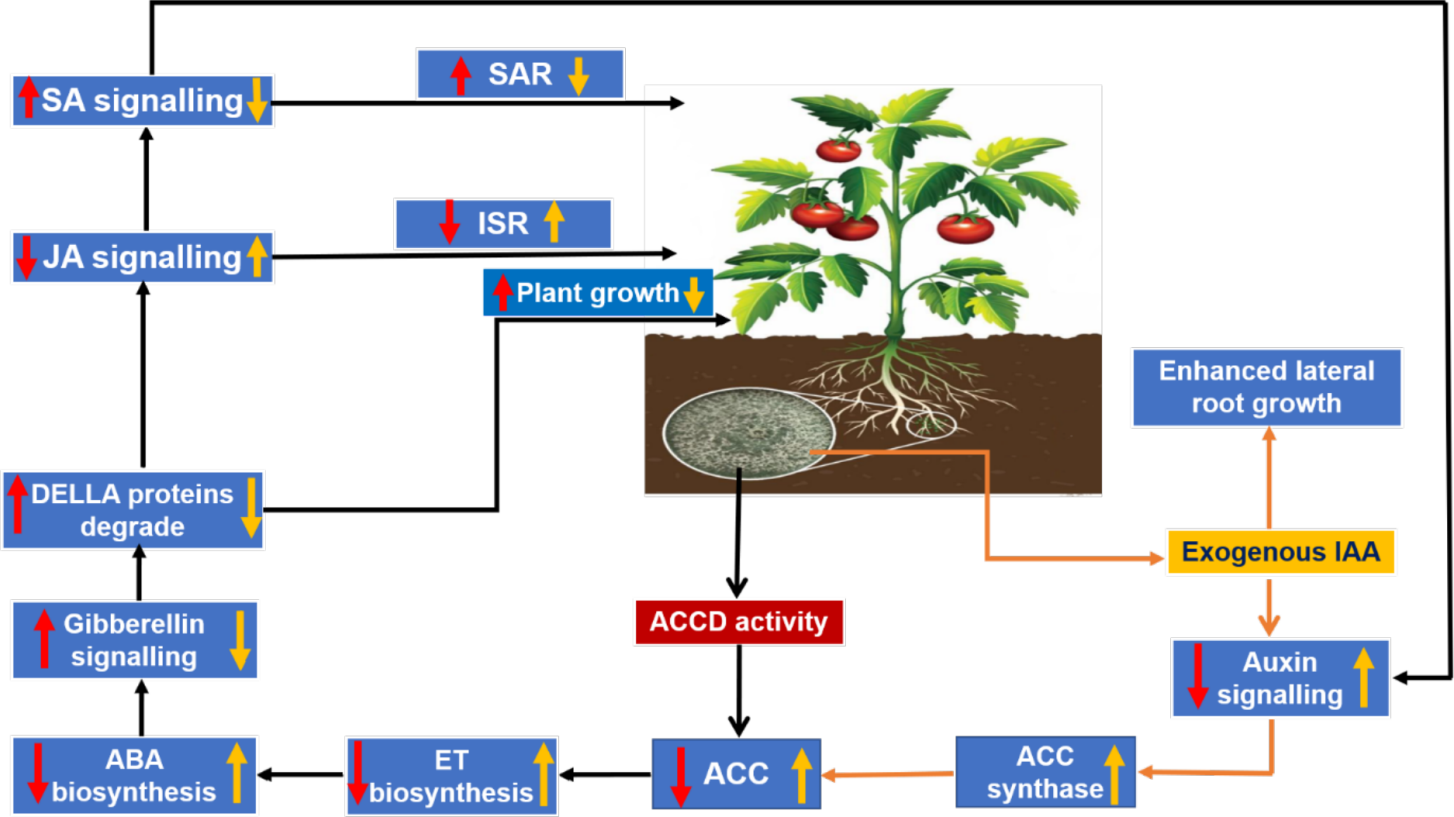
*Trichoderma*–plant cross-communication model *via* regulation of the phytohormonal blend: red arrows indicate the effects on the plant due to 1-aminocyclopropane-1-carboxylate deaminase (ACCD) activity in *Trichoderma*; and yellow arrows indicate the modulation in phytohormonal concentration due to exogenous production of indole acetic acid (IAA) by *Trichoderma*.

The phytohormonal blend produced by plants in response to root colonization by *Trichoderma* also plays an important role in determining plant growth and development. A balanced trade-off between growth and defense in plants upon colonization by *Trichoderma* can be explained in terms of cross-communication among phytohormones, viz., ET, SA, and JA (the central players of defense); abscisic acid (ABA), which is related to abiotic stress and plant growth; indole acetic acid (IAA), which is commonly associated with plant growth and lateral root growth of plants; and gibberellins, which modulate plant growth and defense responses *via* degradation of the DELLA protein (D, aspartic acid; E, glutamic acid; L, leucine; L, leucine; A, alanine) (Hermosa et al., 2012). During *Trichoderma*–plant interactions, the 1-aminocyclopropane-1-carboxylate deaminase (ACCD) activity of *Trichoderma* reduces ET production by lowering the availability of the substrate 1-aminocyclopropane 1-carboxylic acid (ACC), which is necessary for ET biosynthesis. As a result, ABA biosynthesis decreases and the activation of gibberellin signaling takes place *via* degradation of the DELLA protein, which results in an increase in PGP activities. Moreover, JA- and SA-mediated defense responses in plants are also modulated by gibberellin through regulation of DELLA protein degradation. Furthermore, IAA and ET can reciprocally regulate biosynthesis of each other (Stepanova et al., 2007 *)* and, according to this finding, ABA biosynthesis is regulated by exogenous auxin-stimulated ET biosynthesis *via* ACC synthase (Hermosa et al., 2012). A decrease in ABA biosynthesis is inversely proportional to stomatal conductance, thereby ensuring a higher rate of photosynthesis, and *vice versa*. Tucci et al. (2011) observed an increase in PGP activity in tomato plants subjected to treatments containing *T. atroviride* and *T. harzianum* (Dutta and Das, 1999; Dutta and Das, 2002). The probable reasons for a reduction in ET production were suggested to be either a decrease in the precursor ACC through microbial degradation of IAA in the rhizosphere or the presence of ACCD activity in *Trichoderma* (Tucci et al., 2011). *T. asperellum* mutants with RNA interference (RNAi) silencing of the ACCD gene showed an inability to promote root elongation in treated canola seedlings, suggesting the important role of ACCD in root elongation and development (Viterbo et al., 2010; Kubicek et al., 2011). Exogenous production of IAA by *Trichoderma* stimulates ET biosynthesis through ACC synthase (**Figure 5**). Liu et al. (2021) conducted an experiment to identify the growth-promoting effect of the *T. guizhouense* NJAU 4742 strain on cucumber seedlings in a hydroponic study. They observed a significant increase in plant biomass and the modification of lateral root architecture, with a 64.7% increase in lateral root tips of treated plants compared with control. Further study on *in situ* biosynthesis of auxin by *T. guizhouense* during interaction with cucumber roots revealed a gradual increase of auxin in the growing media, which was 1.15 and 0.5 times more than the control and IAA-containing treatments (external source) at 30 days post inoculation. These findings indicate that, after interaction with host roots, the exogenous production of IAA by *Trichoderma* increased considerably, which could be the underlying reason behind plant growth promotion. Similarly, Dutta et al. (2021) observed that groundnut plants treated with a *T. harzianum*-based bioformulation, made from a native isolate of Meghalaya, were not only protected from tikka disease but also exhibited enhanced plant growth parameters along with increased lateral root growth and root nodulation. Thus, the role of the phytohormonal blend resulting from *Trichoderma* colonization in determining plant growth promotion and immune response cannot be denied. Therefore, it can be said that plant root colonization by *Trichoderma* and the existence of *Trichoderma* within the plant as an endophyte (Zheng et al., 2021), which stimulate the plant’s immunity responses, constitute a complex yet profitable relationship that enables the plant to withstand subsequent biotic and abiotic stresses.

## 4 Soluble secretome of *Trichoderma*: role in host–plant interaction and biological control of plant diseases

**Figure 6:**
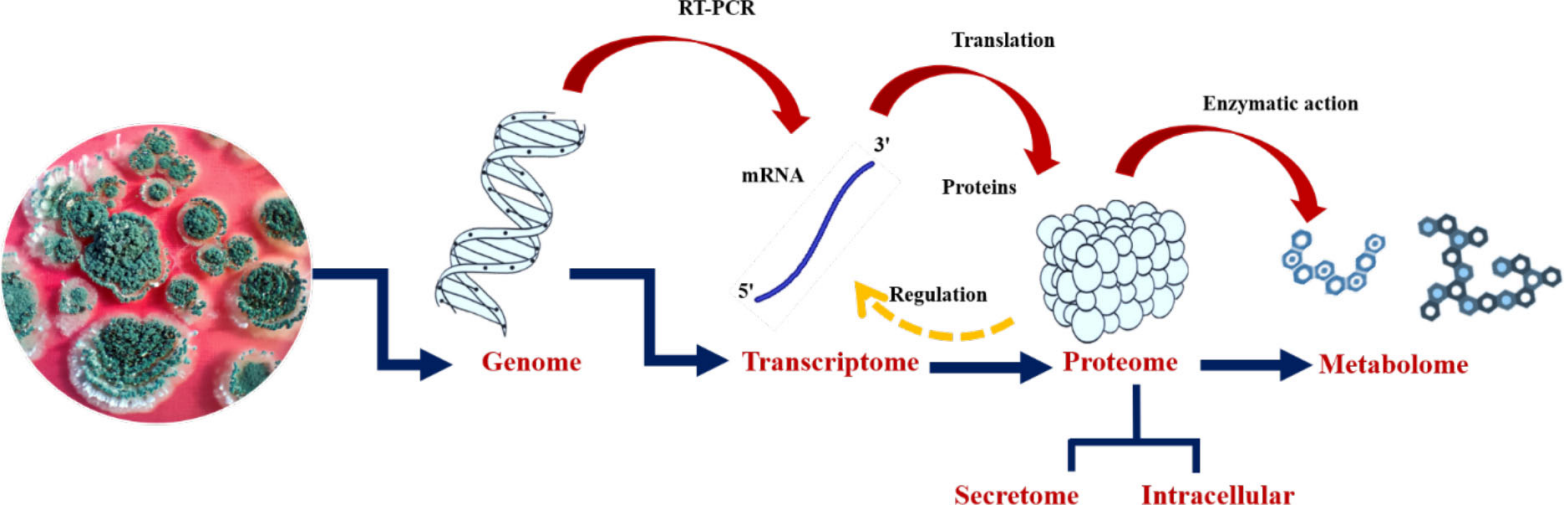
Omics strategies for a better understanding of molecular dialogues in *Trichoderma*–plant/phytopathogen interaction.

The emergence of the era of molecular science in the 1940s and 1950s, and its subsequent progress, with the development of different biotechnology tools, made it possible for scientists to isolate, study, and determine the chemical composition of individual genes present in any organism, and ultimately paved the way for whole-genome sequencing. The ability to map and study genes present in a genome made it easier for scientists to understand how genes are assembled in a genome and how they perform their function. In this context, the development of evolutionary trees was also fine-tuned by the detailed knowledge obtained from the understanding of genomics (Merrill and Mazza, 2006). The comprehensive study or global assessment of a set of molecules is referred to as “omics”. Next-generation sequencing of genetic materials and the development of other high-throughput technologies has led to availability of omics data worldwide. The first omics to appear was genomics, which deals with the study of the whole genome of an organism (Hasin et al., 2017). Different omics, viz., genomics, transcriptomics, proteomics, and metabolomics, contribute to the wealth of omics data available publicly across the globe (**Figure 6**). *Trichoderma reesei* was the first species of *Trichoderma* to have its whole genome sequenced (Martinez et al., 2008). Subsequently, the complete genome sequencing of many species of *Trichoderma* was carried out, and the genomic information is available from the NCBI (National Center for Biotechnology Information) GenBank database (**Table 2**).

**Table 2:** Genomic information for different *Trichoderma* species available from the NCBI GenBank database.

Species name	Clade under Hypocreaceae	Genome size (Mb)	Total number of genes	Reference
*T. atroviride*	Clade Viridae	36.1	11,863	Kubicek et al. (2011)
*T. atrobruneum*	Clade Harzianum	39.15	8,649	Fanelli et al. (2018)
*T. arundinaceum*	Clade Brevicompactum	36.87	10,473	Proctor et al. (2018)
*T. asperellum*	Clade Viridae	37.66	12,586	Druzhinina et al. (2018)
*T. citrinoviride*	Clade Longibrachiatum	33.2	9,737	Druzhinina et al. (2018)
*T. gamsii*	Clade Viridae	37.9	10,709	Baroncelli et al. (2015)
*T. guizhouense*	Clade Harzianum	38.8	11,297	Druzhinina et al. (2018)
*T. hamatum*	Clade Viridae	38.2	12,391	Studholme et al. (2013)
*T. harzianum*	Clade Harzianum	40.9	14,095	Druzhinina et al. (2018)
*T. koningiopsis*	Clade Viridae	36.58	12,661	Castrillo et al. (2017)
*T. longibrachiatum*	Clade Longibrachiatum	31.7	9,409	Xie et al. (2014)
*T. parareesei*	Clade Longibrachiatum	32.07	9,292	Yang et al. (2015)
*T. reesei*	Clade Longibrachiatum	34.1	9,129	Martinez et al. (2008)
*T. virens*	Clade Virens	39.0	12,427	Kubicek et al. (2011)

Sanger expressed sequence tag (EST) projects have made transcriptomics studies of *Trichoderma*–plant interactions easier (Steindorff et al., 2012; Silva et al., 2019). In addition, a transcriptomic study of *Trichoderma* genes present in the fungal cell wall can be obtained using high-throughput techniques such as suppression subtractive hybridization (SSH) (Vieira et al., 2013). Furthermore, a combined omics strategy can be adopted for an in-depth study of *Trichoderma*–plant/pathogen interactions (**Figure 6**).

## 5 Omics in determining the adaptation behavior of *Trichoderma* to host root colonization

*Trichoderma*, being a beneficial microbe, has attracted the attention of researchers, who have studied how it adapts to plant root colonization. The upregulation of genes responsible for the formation of infection structures was observed in an early transcriptomic study of *Trichoderma* colonizing tomato roots in a hydroponic system (Samolski et al., 2009). Similarly, Lawry (2016) observed the presence of an appressorium-like structure in a *Trichoderma virens*–maize hydroponic system that helped the biocontrol agent penetrate the plant cell wall and form an intercellular infection peg. The infection pegs possess structures similar to a haustorium, thereby indicating intracellular colonization of maize roots by *T. virens*. Moreover, forced penetration, by pushing away the outer cell layer of the epidermis, was observed in maize grown in *T. virens*-inoculated soil, and may be considered as another root colonization mechanism of *Trichoderma*. Rubio et al. (2014) reported a decrease in nutrient and carbohydrate metabolism activity in plants in response to hyphal attachment of *Trichoderma*. After 20 h of interaction between *Trichoderma* and tomato grown in a hydroponic system, Rubio et al. (2012) observed the differential regulation of genes in *Trichoderma* as a response to the host’s fluctuating behavior. It revealed an upregulation of *Trichoderma* genes involved in carbohydrate metabolism, nutrient exchange with the plant, the generation of building blocks, and cell wall synthesis, and a downregulation of genes that indicate a sufficient availability of nitrogen. Furthermore, the upregulation or downregulation of genes in *Trichoderma* during root colonization of either tomato or maize plants is greatly regulated by the host itself. In a *T. virens*–maize co-culture system, the genes that are mostly upregulated belong to classes such as the glycosyl hydrolases (GHs), oxidoreductases, and small secreted proteins, and the symbiosis-related invertase TvInv (Vargas et al., 2009). The gene most preferentially expressed in *T. virens* during its interaction with tomato was revealed to be a secreted quino-protein, glucose dehydrogenase. Several studies on the secretome of different species of *Trichoderma* co-cultured with plants have reported an increased expression of genes encoding glycosidases and peptidases during the initial phase of their interaction (Lamdan et al., 2015; González-López et al., 2021). Reduction in tomato root colonization by a *T. harzianum* mutant with a silenced *thpg1* gene encoding endopolygalactouronase indicates the probable role of fungal glycosidases and peptidases in plant–*Trichoderma* interactions. Therefore, it can be said that the lytic enzymes released by *Trichoderma* during the initial phase of their interaction with plants is essential for disintegrating cell wall components to aid in the successful colonization of plant roots (González-López et al., 2021). Furthermore, during the first 24 h of interaction, the genes encoding a group of antioxidant enzymes were found to be exclusively expressed, which is necessary for ROS detoxification, and therefore demonstrates *Trichoderma*’s strategy of adaptation to the highly reactive environment of plants (González-López et al., 2021). However, there is still a need to perform more function-oriented experiments to obtain a clear understanding of the biochemical significance of host specificity at a transcriptome level. Overall, it can be summarized that the host and *Trichoderma* appear to coordinate their counterattacks, and *Trichoderma* has several genes for secreted proteins that apparently play a key role in determining its ability to survive in the complex rhizosphere ecosystem.

Proteomic study of the secretome of *Trichoderma* revealed that 3.5%–6.0% of total proteins are released *via* the type II secretion system into the apoplastic space of the plant cell, which is also known as the soluble secretome of *Trichoderma*. Gene ontology studies of the soluble secreted proteins of *Trichoderma* have revealed the range of different functions exhibited by these proteins, which are primarily CWDEs, cell wall adherence proteins (such as adhesins, hydrophobins, and tandem repeats), effector-like proteins, proteins determining host surface attachment and recognition, and proteins involved in secondary metabolisms (Mendoza-Mendoza et al., 2017). An effort is made here to briefly discuss the literature available on the soluble secretome of *Trichoderma* with regard to plant root colonization and its role in the biological control of plant diseases.

## 6 Cell wall-degrading enzymes

In every plant–microbe interaction, the host cell wall is at the forefront and so is the basal defense. Therefore, it is essential that the micro-organisms digest cell walls by releasing CWDEs in order to break through to the host system. Plant CWDEs are considered a major source of communication molecules and belong to CAZymes groups. GHs essentially digest plant cell walls and facilitate fungal entry into the host tissue. A study conducted with different species of *Trichoderma* revealed that *T. harzianum* and *T. guizhouense* secrete a higher number of CAZyme modules than other *Trichoderma* species. This may be indicative of their larger genome size and/or their particular behavior in the competitive environment of soil and when interacting with plants and pathogens (Li et al., 2013). In soil, *Trichoderma* releases a lignocellulolytic enzyme that helps it to live a saprophytic life. However, in the presence of host root exudates they respond differently, synthesizing and secreting CWDEs (Cragg et al., 2015). Harman et al. (2004) reported two glucosyltransferases (GTs) from the soluble secretome of mycoparasitic fungi *T. virens* and *T. atroviride.*

The soluble secretome of *Trichoderma* contains a significant amount of CWDEs. They are responsible for alerting the plant immune system to the presence of an invader. As reported by Avni et al. (1994), inactive cellulase and xylanase were the first MAMPs obtained from *Trichoderma*. Furthermore, CWDEs cause damage to the plant cell wall, thereby generating DAMP signals. A study of *T. harzianum*–root interaction in *Arabidopsis* and tomato led to the discovery of the first DAMP, which corresponds to the oligogalactouronides produced by the enzymatic activity of CWDE endopolygalacturonase ThPG1 in *T. harzianum*, which was found to be capable of inducing systemic defense in the plants (Moran-Diez et al., 2009). In a study conducted by Baroncelli et al. (2016), the expression of two endo-polygalcturonase genes (viz., *TvPg1* and *TvPg2*) from *T. virens* I10 was examined during the interaction with tomato roots. The results revealed that, while interacting with, in particular, host roots or pectin, expression of *TvPg1* was induced, whereas *TvPg2* was later expressed constitutively. According to Sarrocco et al. (2017), this constitutively produced endopolygalacturonase was responsible for eliciting ISR in the host. Similarly, the direct activity of plant chitinases or the mycotrophic nature of *Trichoderma* against rhizospheric fungi yields chito-oligosaccharides, which can also function as DAMPs in the activation of systemic plant immunity (Woo et al., 2006). Apart from inducing plant immunity by generating MAMP and DAMP signals, CWDEs also play an important role in determining the efficient colonization of roots by *Trichoderma*. They achieve this by either increasing the plasticity of the host cell wall or causing irreversible deterioration of the cell wall structure.

As the fungal cell wall comprises mainly chitin, glucan, and proteins, mycoparasitism by *Trichoderma* involves the extensive use of CWDEs, viz., chitinases, glucanases, and proteases (**Table 3**). Use of a *Trichoderma* microarray to study the transcriptomic changes of genes in *T. atroviride* overgrown on a *Verticillium dahliae* colony revealed that there was total 143 differentially regulated genes (almost 98%) that belonged to the *T. atroviride* genome. The upregulated genes were all from classes of CAZymes and proteases, viz., serine, aspartic acid, and metallopeptidases genes that are crucial in weakening and disintegrating the fungal host cell wall (Morán-Diez et al., 2019). Therefore, it can be said that the differentially regulated genes of *T. atroviride* are unequivocally associated with the mycoparasitic and antagonistic activity against the targeted pathogen.

**Table 3:** Enzyme profile of *Trichoderma* in mycoparasitic interactions.

Enzyme group	Enzyme name	Molecular weight (kDa)	Reference
Chitinases	Endoquitinase	33–37	Ulhoa and Peberdy (1991); De La Cruz et al. (1992); Harman (1993)
	Endoquitinase	52	Harman (1993)
	Endoquitinase	31–33	De La Cruz et al. (1992)
	Endoquitinase	46	Lima et al. (1997)
	Exoquitinase	40	Harman (1993)
	*N*-acetylglicosaminidase	102–118	Ulhoa and Peberdy (1991)
	*N*-acetylglicosaminidase	73	Harman (1993); Lorito et al. (1994)
	Exochitinase 1	–	Pellan et al. (2021)
	Exochitinase 2	–	
Glucanases	Endoglucanase (EG Th1)	23.5	Liu et al. (2013)
	Exoglucanase (ExG Th1)	61	
	Endo-1,3-β-glucanase	76	Lorito et al. (1994)
	Endo-1,3-β-glucanase	36	De La Cruz et al. (1995)
	Endo-1,3-β-glucanase	40	Noronha et al. (2000)
	Exo-1,3-β-glucanase	29	
	Endo-β-1,6-glucanase	46	Monteiro and Ulhoa (2006)
	Exo-1,3-β-glucanase	78	
	Exo-1,3-β-glucanase	110	Cohen-Kupiec et al. (1999)
	Exo-β-1,3-glucanase	83.1	Bara et al. (2003)
	α-1,3-Glucanase MUT1 (MutAp)	–	Grun et al. (2006)
	Endo-β-1,3-glucanase	–	Suriani Ribeiro et al. (2019)
	β-1,3-Glucanase	–	Senthilkumar et al. (2021)
Other enzymes from the *Trichoderma* secretome	α-Mannosidase	53.52	Monteiro et al. (2010)
	Acid phosphatase	41.71	
	α-1,3-Glucanase	71.79	
	Carboxypeptidase 2^a^	53.79	
	Glucosidase I	27.50	
	α-Mannosidase	53.52	
	Carboxypeptidase 2^b^	53.45	
	Endochitinase	41.71	
	Aspartate protease	–	Ramada et al. (2016)
	Serine protease	–	
	Trypsin-like protease	–	
	Endo-β-1,4-glucanase	–	
	β-Endo-1,3-glucanase	–	
	α-1,3-Glucanase		
	α-1,2-Mannosidase	–	
	α-l-Arabinofuranosidase	–	
	α-Galactosidase	–	
	β-1,6-Glucanase	–	
	Endo-1,3(4)-β-glucanase	–	
	Endochitinase chit33	33	
	chit37 Endochitinase	37	
	chit42 Endochitinase	42	
	β-1,3-Exoglucanase	107.28	Kohler and Tisserant (2014)
	Neutral metalloprotease *NMP1*	–	Zheng et al. (2016)
	β-1,3-Exoglucanase	107.93	Blauth de Lima et al. (2017)
	Endochitinase	42	
	Endochitinase	34.026	
	Glucoamylase	66.25	
	Mutanase	67.63	
	Serine endopeptidase	42.47	
	β-Glucocerebrosidase	51.59	Nauom et al. (2019)
	β-1,3-Glucanase	40.1	
	1,4-α-Glucosidase	67.28	
	α-d-Galactosidase	48.25	
	1,2-α-Mannosidase	55.65	
	Peptidase M14	46.95	
	Endo-1,3-β-glucanase	92.19	
	Tyrosinase	46.95	
	Peptidase S8	92.55	

## 7 Enzymes for chitin degradation

It has been reported that the *Trichoderma* genome harbors a greater number of genes encoding chitinolytic enzymes, which is attributed to *Trichoderma*’s mycoparasitic nature. Fungal chitinases belong to the GH18 and GH20 families. GH18 chitinases can be further categorized into subfamilies A, B, and C. It has been observed that genes encoding chitinases from GH18 are significantly expanded in *T. atroviride*, *T. asperellum*, *T. atrobrunneum*, *T. gamsii*, *T. harzianum*, and *T. virens* (Kubicek et al., 2011). Chitin and chitosan (a partial or complete deacetylated derivative of chitin) comprise the chitinous layer of fungal cell wall. Chitosan in mycoparasitic fungi such as *Trichoderma* plays an important role in the scavenging of ROS produced by parasitized fungi. Kappel et al. (2020) observed that out of six genes encoding chitin deacetylase, the deletion of genes *cda1* or *cda5* in *T. atroviridae* led to severely impaired mycoparasitic ability. This result indicates that a decrease in or absence of chitin deacetylase enzymes results in a low level of chitosan in *Trichoderma*, and, therefore, the *Trichoderma* is not protected from ROS (Mukherjee et al., 2022). Further study led to the discovery of cell wall remodeling in *T. atroviridae* during mycoparasitic interaction *via* the upregulation of all six genes encoding chitosanase, especially toward the later stage of interaction. One interesting finding made during this study by Kappel et al. (2020) was CHS8, which is called as a hybrid synthase due to its similarity to both chitin synthases and hyaluronan synthases, and can utilize both UDP-*N*-acetylglucosamine and UDP-d-glucuronate as substrates. The authors, therefore, speculated that CHS8, along with CDA1, forms a chitin glycol-polymer layer that protects the *Trichoderma* cell wall during mycoparasitic interactions (Kappel et al., 2020).

## 8 Glucan degradation

The enzymes α- and β-glucanase are necessary for the deconstruction of the glucan layer. Fungal α-1,3-glucanases are members to the GH71 family. *T. harzianum* and *T. asperellum* explicitly secrete the exo-α-1,3-glucanases AGN13.1 and AGN13.2, respectively, in the presence of the *Botrytis cinerea* cell wall. Enzyme AGN13.1 is found to possess lytic properties against fungal cell walls and exhibit antifungal activity (Mukherjee et al., 2022).

β-1,3-Glucanases are classified into GH families, viz., 16, 17, 55, 64, and 81. The mycoparasitic *Trichoderma* genomes comprise a large number of genes encoding GH55 and GH64 family members (Kubicek et al., 2011). They play a significant role in the mycoparasitization of oomycete fungi (which have a cell wall composed of cellulose and β-1,3- and β-1,6-glucans). A study of *T. virens* mutants in which the *bgn3* gene, encoding β-1,6-glucanase, is overexpressed found that such mutants exhibited enhanced antagonism toward *Globisporangium ultimum*, whereas mutants overexpressing the genes for both β-1,3-glucanase and β-1,6-glucanase were found to exhibit enhanced inhibition of *G. ultimum* (Djonovic et al., 2007).

## 9 Protein degradation

Proteases are an important group of enzymes released by *Trichoderma* in the event of mycoparasitism. The differential regulation of several protease genes of *Trichoderma* is reported during mycoparasitism (Mukherjee et al., 2022). Overexpression of the *T. atroviride prb1* gene (encoding protease) reportedly provides increased protection against *R. solani* (Pozo et al., 2004). Cortes et al. (1998) observed that expression of the *prb* gene was induced before contact with the fungal host. Further study of gene behavior in nitrogen-limited conditions led to the finding that the promoter region of the *prb* gene contains a binding site for transcriptional activator of nitrogen catabolite-repressed genes, viz., *ARE1* (Olmedo-Monfil et al., 2002; Mukherjee et al., 2022). These findings caused Druzhinina et al. (2011) to hypothesize that, in the early stage of mycoparasitic interaction, the activity of proteolytic enzymes results in host-derived nitrogenous products, which are responsible for the activation of mycoparasitism-relevant genes *via* their binding to nitrogen sensors present on the *Trichoderma* cell surface.

## 10 Small cysteine-rich proteins as effectors of *Trichoderma*

Effectors released during *Trichoderma*’s interactions with plants or fungi participate in ROS scavenging, chitinase and glucanase production, fungal cell wall masking, protease inhibition, and the prevention of defense alarm activation in neighboring cells colonized by the invader (Rabe et al., 2013; Lanver et al., 2017). A study conducted by Mendoza-Mendoza et al. (2017) revealed the presence of 70–123 effector proteins in the soluble secretome of *Trichoderma*. However, not all these effectors possess a clear functional domain. Some of the effector proteins with known functional domains are discussed briefly herein.

## 11 Common in fungal extracellular membrane domain proteins

Common in fungal extracellular membrane is a protein domain containing eight cysteines, which distinguishes it characteristically from other known cysteine-rich proteins. CFEM domain proteins were first discovered in rice blast pathogen *Magnaporthe grisea* (Kulkarni et al., 2003) and are known to play important roles in fungal pathogenicity. The functions of CFEM domain proteins include plant-surface sensing, appressorium development, asexual development (Sabnam and Barman, 2017), iron assimilation (Nasser et al., 2016), and redox homoeostasis (Kou et al., 2017). Fifty soluble secreted proteins of *Trichoderma* have so far been found to contain CFEM domains.

In an experiment on a *Trichoderma*–maize co-culture conducted in a hydroponic system, a decreased abundance of several CFEM-containing secreted proteins was observed. On developing deletion mutants for two genes encoding CFEM domain proteins with IDs 92810 and 111486 (Joint Genome Institute (JGI) v2.0), Lamdan et al. (2015) observed an increased ISR response against necrotic phytopathogens. They suggested that an increased degradation or sequestering of CFEM domain proteins by host roots could be the reason for their loss of abundance in a *Trichoderma*–maize co-culture system. However, further study of CFEM domain proteins is needed to reveal their modeof action in *Trichoderma*–plant *or Trichoderma–*phytopathogen interactions.

## 12 LysM-like putative effectors

Chitin, a homopolymer of *N*-acetyl-d-glucosamine, represents the second most abundant organic matter after cellulose. Chitin is widely distributed in fungi as a major component of the cell wall, but is absent in plants. The presence of chitin in the plant system is recognized by specific lysin motif (LysM)-containing pattern recognition receptors (PRRs) in the plant cell surface, which trigger an innate immune response in plants (Marshall et al., 2011). The absence of these PRRs compromises the plant’s defense against fungal pathogens. Therefore, the plant’s ability to perceive chitin is very important in recognizing phytopathogenic fungi. However, successful plant colonizers have evolved strategies that overcome chitin-induced defense in plants. Alteration of cell wall composition and release of LysM-like effector proteins are some of the strategies adopted by micro-organisms. LysM-like effectors released by plant colonizers bind to the free chitin released in the plant apoplastic space during fungal growth and mask the colonizer’s presence. In this way, they overcome chitin-induced plant defense. Genomic study of mycoparasitic and endophytic *Trichoderma* has revealed that they contain an increased number of genes encoding LysM-containing secreted and non-secreted proteins as well as chitinases. These proteins help in the penetration and establishment of *Trichoderma* within the plant system by binding themselves to the fungal chitin and thereby avoiding ligand–PRR binding (Hermosa et al., 2013). Moreover, it has also been suggested that proteins containing a LysM domain may provide a mechanism of self-protection against the *Trichoderma*’s own chitinases (Gruber and Seidl-Seiboth, 2012).

## 13 Hydrophobins

Hydrophobins are small, unique, surface-active fungal proteins with the ability to form an amphipathic membrane at the interface of hydrophilic and hydrophobic environments. Their β-structured core is composed of eight highly conserved cysteine residues linked by four disulfide bridges. Hydrophobin proteins have a large exposed hydrophobic area, which explains their high surface activity (Linder et al., 2005; Bayry et al., 2012). Class I hydrophobin molecules form rodlet layers on the fungal cell wall by organizing themselves into a highly insoluble amphipathic membrane at the junction of the hydrophilic fungal cell wall and the hydrophobic environment. Class II hydrophobins form micro-aggregates to give rise to dimers and tetramers in a rodlet-like structure (Mendoza-Mendoza et al., 2017). Kubicek et al. (2008) carried out a comparative evolutionary study on class II hydrophobins produced by the ascomycetes group of fungi and noted that the genus *Trichoderma* ranked first in number and diversity of class II hydrophobins. Guzman-Guzman et al. (2017) reported that a class II hydrophobin, viz., TVHYDII1 of *T. virens*, contributes to the antagonistic activity of *T. virens* against *R. solani* and promotes *Arabidopsis* root colonization by *Trichoderma*. Huang et al. (2015) observed an up-regulation in hydrophobin synthesis and secretion in *T*. *asperellum* when placed in a 1% *Alternaria alternata* cell wall and 5% *A. alternata* fermentation broth, which is indicative of hydrophobins’ role in mycoparasitism. Microarray analysis of *T. virens T87* genes revealed that genes encoding hydrophobins were largely downregulated during *Trichoderma*–tomato interaction (Rubio et al., 2012) and found to have a negative effect on the growth and development of tomato plants in *in vitro* conditions. This result may be indicative of limited root attachment of *T. virens T87* due to fewer hydrophobins, affecting its interaction with tomato plants. Interestingly, Przylucka et al. (2017) have observed upregulated *HFB7* genes of *T. virens* in interactions with tomato. *T. harzianum* secretes QID74, which is a hydrophobin-like cell wall protein with a high molecular mass. It is particularly involved in fungal cell wall protection, adherence to the host cell, and modification of the host root architecture by increasing lateral roots growth, which, in turn, ensures increased nutrient uptake. Effective utilization of these nutrients results in increased plant biomass, and promising results have been obtained in cucumber and tomato plants (Samolski et al., 2012).

## 14 Ceratoplatanin family proteins

Ceratoplatanins (CPs) are non-enzymatic unique fungal proteins similar to plant expansin proteins. *Trichoderma* CPs bind to chitin (Pazzagli et al., 2014), which may be helpful in opening the physical spaces of the parasitized fungal cell wall. Similarly, during colonization of the host plant, the behavior of CP proteins might be helpful in masking fungal cell wall chitin from detection by the host plant’s receptors (Quarantin et al., 2016). CPs are also known as eliciting plant response-like proteins (EPLs) due to their role in the induction of SAR in plants (Gaderer et al., 2014). According to Gao et al. (2020), the number of EPL-encoding genes in *Trichoderma* species is either three or four. During *T. harzianum–S. sclerotiorum* interaction, the role of EPL1 was found to be significant for the expression of genes related to mycoparasitism and coiling around *S. sclerotiorum*. Moreover, EPL-encoding genes found to downregulate the expression of virulence genes present in *B. cinerea* necessary for botrydial biosynthesis. Therefore, EPLs have two major functions in *Trichoderma*–pathogen interaction, viz., the expression of mycoparasitism-related genes and protection of *Trichoderma* from secondary metabolites produced defensively by pathogens (Mukherjee et al., 2022).

In *Trichoderma*, the *Sm1* gene encodes a small, secreted protein belonging to the CP family. *Sm1* gene abundantly expressed throughout fungal development. Specific growth conditions modulate the expression of the *Sm1* gene in *Trichoderma*. Studies conducted by Vargas et al. (2011) concluded that the detection of sucrose-rich plant root exudates and their trafficking activates the expression of *Sm1* in *T. virens*. Djonovic et al. (2006) demonstrated the role of *Sm1* in *T. virens* as a non-enzymatic effector of plant defense. In addition, purified Sm1 protein was observed to trigger ROS production, thereby eliciting local and systemic resistance in the plant. Furthermore, these proteins are found to have no phytotoxic or antimicrobial function. *Trichoderma* has a wide host range, and therefore its interactions with its hosts have diverse consequences. Salas-Marina et al. (2015) observed that *Sm1*- and *Epl1*-deleted mutants of *T. virens* and *T. atroviride* led to decreased systemic resistance in treated tomato plants, and overexpression of these genes resulted in enhanced protection against phytopathogens. In a tripartite interaction involving *T. virens* Gv29-8, maize, and *Colletotrichum graminearum*, Crutcher et al. (2015) observed an enhanced expression of Sm2 protein by *T. virens.* The use of mutants developed by deletion of the Sm2-encoding gene revealed that they were able to induce same level of ISR in maize plants; however, the root colonization ability of *T. virens* was found to decrease significantly. Conversely, a study on the interaction of *Sm1*-deleted mutants of *T. virens*, I10 maize, and *Cochliobolus heterostrophus* revealed a decreased level of ISR, and an *Sm1*- and *Sm2*-deleted mutant caused a more severe reduction in the plant’s defense response (Gaderer et al., 2015). The diverse responses obtained from these tripartite interactions could be due to the different lifestyles of the phytopathogens used for the study. The plant’s immune response to biotrophs, hemibiotrophs, and necrotrophs upon infection could determine which *Trichoderma* elicitors/effectors are deployed. Indeed, the activity of the same *Trichoderma*-derived effector could be altered by the host plant’s response, thereby explaining some of the distinct actions of *Sm1* and its paralogs depending on the three-way interaction.

## 15 Swollenin

Swollenin is a soluble secreted protein first described in *T. reesei*. Swollenin and its orthologs are structurally characterized by the fungal carbohydrate-binding domain (CBD) in their N-terminal, which is followed by a region with domains 1 and 2, similar to plant expansin (Saloheimo et al., 2002). The presence of CBD in their N-terminal helps them to bind to the carbohydrate molecules present in the plant cell wall, and facilitates access to and colonization of the plant system. Brotman et al. (2008) observed that TasSwo—a swollenin protein secreted by *T. asperellum*—recognizes and binds to the cellulose present in the plant cell wall *via* the CBD and alters the architecture of the plant cell wall in favor of root colonization by *Trichoderma*. Furthermore, the authors demonstrated that the CBD present in these proteins acts as a MAMP by inducing plant-innate immunity in cucumber to phytopathogens such as *Botrytis cinerea* and *Pseudomonas syringae*.

## 16 Wall stress responsive-component domain protein

Genomic study of *Trichoderma* has revealed the presence of several proteins with the cell wall stress-responsive component (WSC) domain. Although no direct information is yet available on how these proteins help *Trichoderma* in their interactions with plants or pathogens, similar proteins encoded in *Trichoderma* genome are also reported in other plant-beneficial endophytes. For instance, in *Piriformospora indica*, the WSC domain protein FGB1 performs the function of plant immunity suppressor by altering its cell wall composition and properties, and therefore aids its establishment within the host plant (Wawra et al., 2016). Moreover, as reported by Tong et al. (2016), these proteins may have a role in promoting cellular resistance, cell wall disruption, high osmolarity, the production of metal ions (Mg^2+^, Zn^2+^, Fe^2+^, Ca^2+^, Mn^2^+, and K^+^), and oxidation (Silva et al., 2019). In addition, under stressed conditions, WSC domain proteins, viz., FGB1 and WSC3, may be involved in β-glucan remodeling in the fungal cell wall (Wawra et al., 2019). A comparative secretome analysis of *Trichoderma* under salt stress conditions revealed that, in the presence of its plant host, the expression of WSC domain proteins in *Trichoderma* decreases, which may be indicative of the benefits derived by the fungus from its symbiont (i.e., the plant) through intensified root colonization (Rouina et al., 2022).

Other than the above-discussed secreted proteins, there are certain proteins identified in *Trichoderma* genomes, the function of which are not yet known. The presence of genes for different proteins, e.g., necrosis-inducing polypeptides (NPP1), killer-like toxins, GLEYA adherence proteins, and fungal ribonucleases (RNAses), are reported in different *Trichoderma* genomes. The expression of some killer-like toxin protein-encoding genes, such as *KP4*, hinders plant growth (Allen et al., 2008); however, as reported by Allen et al. (2011), the inclusion of this gene in transgenic plants is effective in protecting the plants from phytopathogens. Similarly, across *Trichoderma* species, the number of secreted GLEYA adhesin protein differs. According to previous studies, *T. guizhouense* harbors the maximum number of such proteins, i.e., three; *T. atroviride*, *T. gamsii*, and *T. parareesei* secrete two proteins; and *T. harzianum*, *T. virens*, and *T. reesei* contain only one such protein. The presence of necrosis-inducing proteins (NPP1) in both mycoparasitic and saprophytic *Trichoderma* genomes and their role in plant interaction is still not clear. However, it has been speculated that *NPP1* genes and their expression are not always related to necrosis in the host plant, but may also play a role in fungal growth and sporulation (Santhanam et al., 2013). Recently, the use of RNA-interacting proteins and RNA by fungi has been reported in the establishment of successful interactions with the host plant (Spanu, 2015). Among three different families of RNAses present in fungi, viz., non-specific RNases, RNase T1, and RNase T2, *Trichoderma* genomes are reported to express two RNAse families, i.e., RNase T2 and non-specific RNases. Although the T2 family RNases are known to perform functions such as nutrient acquisition, phosphate solubilization, defense against phytopathogens, self-incompatibility, and senescence (Deshpande and Shankar, 2002); however, their role in establishing interaction with plants is still not known.

## 17 Conclusion

*Trichoderma* is widely used across the globe due to its biocontrol and plant growth-promoting abilities. The interactions of *Trichoderma* spp. with host plants and pathogens at a molecular level will provide insights on the mechanisms that make *Trichoderma* a superior biocontrol agent. Mycoparasitism by *Trichoderma* is a complex process; therefore, a comprehensive study at the gene level is important to understand how the BCA safeguards itself from the defense strategies adopted by the parasitized fungi. Moreover, knowledge of secondary metabolites secreted by *Trichoderma* during their interaction with either the plant host or fungal host may be helpful in formulating effective bioactive molecule-based formulations that can provide enhanced protection to plants for a longer time. An understanding of the molecular dialogues between the host plant/fungus and *Trichoderma* is important to realizing the full potential of *Trichoderma* as a biocontrol agent.

## Author contributions

MM prepared the original draft, and PD reviewed and edited the article. All authors contributed to the article and approved the submitted version.
